# EpCAM and the biology of hepatic stem/progenitor cells

**DOI:** 10.1152/ajpgi.00069.2014

**Published:** 2014-12-04

**Authors:** Laurent Dollé, Neil D. Theise, Eva Schmelzer, Luke Boulter, Olivier Gires, Leo A. van Grunsven

**Affiliations:** ^1^Department of Biomedical Sciences, Liver Cell Biology Lab, Vrije Universiteit Brussel, Brussels, Belgium;; ^2^Departments of Pathology and Medicine, Beth Israel Medical Center of Albert Einstein College of Medicine, New York, New York;; ^3^McGowan Institute for Regenerative Medicine, University of Pittsburgh, Pittsburgh, Pennsylvania;; ^4^Medical Research Council Human Genetics Unit, Institute for Genetics and Molecular Medicine, Edinburgh, Scotland; and; ^5^Department of Otorhinolaryngology, Head and Neck Surgery, Grosshadern Medical Center, Ludwig-Maximilians-University of Munich, Munich, Germany

**Keywords:** EpCAM, liver differentiation, liver regeneration, hepatic stem/progenitor cell, signaling

## Abstract

Epithelial cell adhesion molecule (EpCAM) is a transmembrane glycoprotein, which is frequently and highly expressed on carcinomas, tumor-initiating cells, selected tissue progenitors, and embryonic and adult stem cells. During liver development, EpCAM demonstrates a dynamic expression, since it can be detected in fetal liver, including cells of the parenchyma, whereas mature hepatocytes are devoid of EpCAM. Liver regeneration is associated with a population of EpCAM-positive cells within ductular reactions, which gradually lose the expression of EpCAM along with maturation into hepatocytes. EpCAM can be switched on and off through a wide panel of strategies to fine-tune EpCAM-dependent functional and differentiative traits. EpCAM-associated functions relate to cell–cell adhesion, proliferation, maintenance of a pluripotent state, regulation of differentiation, migration, and invasion. These functions can be conferred by the full-length protein and/or EpCAM-derived fragments, which are generated upon regulated intramembrane proteolysis. Control by EpCAM therefore not only depends on the presence of full-length EpCAM at cellular membranes but also on varying rates of the formation of EpCAM-derived fragments that have their own regulatory properties and on changes in the association of EpCAM with interaction partners. Thus spatiotemporal localization of EpCAM in immature liver progenitors, transit-amplifying cells, and mature liver cells will decisively impact the regulation of EpCAM functions and might be one of the triggers that contributes to the adaptive processes in stem/progenitor cell lineages. This review will summarize EpCAM-related molecular events and how they relate to hepatobiliary differentiation and regeneration.

the liver is one of the most complex and multifunctional organs of the body of vertebrates. Its vital roles range from activities, such as drug metabolism and detoxification of harmful components, to the biosynthesis of hormones and proteins essential for digestion via the decomposition of red blood cells and the storage and metabolism of glycogen. Accordingly, the liver is a central organ, and injury or malfunctions of it are life-threatening situations. Owing to scarcity of liver donors, which hampers both transplantation of whole organs and of cell suspensions, transplantation of hepatic stem/progenitor cells (HSPCs) has been explored as a valuable bridge and legitimate, alternate strategy for whole-organ replacement. HSPCs have the potential to provide patients with a comprehensive liver regeneration, as these cells have the capacity to differentiate in any epithelial cell type of the liver, i.e., hepatocytes and cholangiocytes (91, 126). To this end, detailed knowledge of the biology of HSPCs, including molecular switches involved in processes of HSPC maintenance and differentiation into mature liver cells, is mandatory and currently understudied. One such potential molecular switch is the epithelial cell adhesion molecule (EpCAM), which is highly expressed in HSPCs but becomes lost during differentiation into hepatocytes (37, 152). EpCAM is a transmembrane glycoprotein, which is primarily expressed in simple epithelia, progenitor cells, normal and malignant stem cells, as well as in numerous carcinomas of different origin (5, 94, 146). The functions of EpCAM are numerous and include cell–cell adhesion, proliferation, maintenance of undifferentiated states, as well as regulation of differentiation, migration, and invasion. Owing to its strong expression in carcinoma cells, most of these functions have been described primarily in malignant cells, and formal proof of function and role in normal cells is often lacking. More recently, the dynamic expression of EpCAM has been discussed in the context of tumor progression and in the process of embryonic stem-cell differentiation (30, 40, 41, 82). Here, EpCAM appears to be expressed in primary tumors and overt metastases, whereas intermediates of the metastatic cascade, i.e., circulating and disseminating tumor cells (CTCs/DTCs), might downregulate EpCAM to gain migratory and invasive properties (40). Likewise, human and murine embryonic stem cells express EpCAM to high levels, which are selectively downregulated during differentiation (41, 99). Based on the strong expression in HSPCs and the dynamic expression pattern of EpCAM during liver cell differentiation, this molecule might represent a central target in the field of hepatocellular differentiation and liver regeneration. The present review will discuss recent advances on EpCAM and HSPC biology, as well as the potential role that EpCAM could play in the decision of HSPCs to commit to a particular cell type during a regenerative response.

## UPDATES ON EpCAM RESEARCH

### 

#### The molecular structure of EpCAM.

**Fig. 1.**
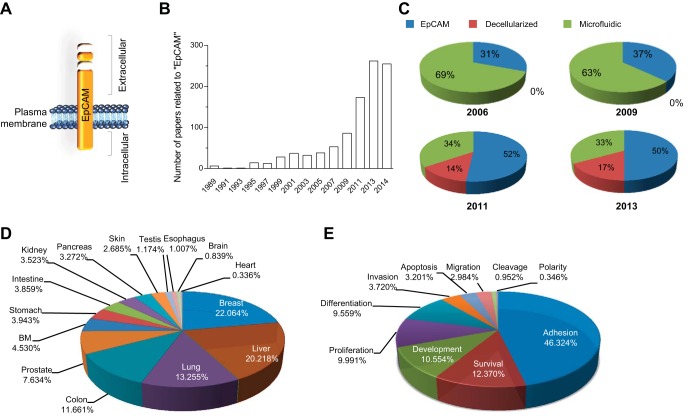
Epithelial cell adhesion molecule (EpCAM) and its significance in research. *A*: schematic representation of the EpCAM structure. *B*: number of papers found nonconsecutively from 1989 to 2014 in PubMed by searching independently the key word “EpCAM.” *C*: repartition in percentage of published papers on EpCAM in combination with 2 other important areas in regenerative medicine: “microfluidic” and “decellularized.” The search was done for 2006, 2009, 2011, and 2013. *D*: percentage of papers describing EpCAM within a certain organ or (*E*) a specific function/role. BM, bone marrow.

EpCAM was first described as an antigen in colon carcinoma cells (52) and is involved in the regulation of normal, malignant, and stem-cell phenotypes (41, 54, 94). However, much of the research on the role of EpCAM in the liver has focused on carcinomas (149, 150). Whereas EpCAM [also known as tumor-associated calcium signal transducer 1, cluster of differentiation 326, or trophoblast cell-surface antigen 1 (TROP1)] was identified as a cell–cell adhesive molecule (75, 77), it does not structurally resemble any of the four major families of cell adhesion molecules (CAMs), namely cadherins (e.g., E-cadherin), integrins (e.g., CD49f), selectins (e.g., E-selectin), and members of the Ig superfamily (e.g., ICAM). Human EpCAM is composed of 314 amino acids (aa) that are divided in three domains: an N-terminal, 242-aa extracellular domain (EpEx); a 23-aa, single-transmembrane domain; and a C-terminal, 26-aa intracellular domain (EpICD; Fig. 1*A*) (115). The sequence of the EpCAM molecule predicts the presence of three N-linked glycosylation sites within the EpEx, which have been experimentally confirmed (20). Glycosylation of EpCAM demonstrates some degree of tissue specificity and was enhanced in tumor vs. normal tissue (104). One possible reason for the enhanced glycosylation of EpCAM in cancers might relate to the positive impact of glycosylation on the molecule's retention time at the membrane (95).

The EpEx further contains EGF and thyroglobulin repeat-like domains, which are involved in both reciprocal interactions between EpCAM molecules on adjacent cells and lateral interactions between EpCAM molecules (4), and comprises 12 highly conserved cysteine residues forming disulfide bonds on the EpEx. The epithelium-specific expression of EpCAM is highly conserved among a variety of vertebrate species, i.e., zebrafish, rodent, dog, and human, suggestive of a central function for EpCAM. For example, murine EpCAM has 81% identity and 89% similarity to the human counterpart.

#### EpCAM research.

The number of papers related to EpCAM has increased markedly since 1997 (Fig. 1*B*). This concerns not only the field of carcinogenesis but also extends to other life-science areas, such as stem-cell biology of the germ-line and somatic cells, morphogenesis, and organogenesis. To acquire a clearer view on the flourishing niche of EpCAM research, we compared the amount of published works on EpCAM within two other well-known protagonists in regenerative medicine (Fig. 1*C*). We chose the “decellularization” technique, a process of tissue treatment by which cells are discharged, but ECM and scaffold remain intact and are now being widely explored for use in the creation of bioartificial organs and “microfluidics” technology, which is the science of designing and manufacturing small devices that deal with tiny volumes of fluid integrated in bioreactors. In 2006, microfluidics was, by far, the most prominent of these research areas, but surprisingly, with time, the proportion of papers related to EpCAM research has increased constantly compared with the two previously described techniques, and from 2010, EpCAM is the most prominent topic within this group (Fig. 1*C*). The increase in publications related to EpCAM is due to two important factors: its expression in epithelial cells in most tissues of the body, which thereby promotes its wide use in various independent areas of research and by many laboratories (Fig. 1*D*), and the discovery that EpCAM is involved in many processes, such as cell adhesion (75), proliferation (83, 96), differentiation (41, 99), migration (30, 110), and cell-cycle progression (18), facilitating its wide expansion and its impact in multiple important research domains (Fig. 1*E*).

Furthermore, due to the prevalence of EpCAM on many carcinomas, it has been (re)discovered many times and has been ascribed >20 different names (115, 129). Alternative nomenclatures are typically derived from the name of the tool used to characterize/identify the antigen: either based on MAb (such as 17/1A or HEA125) or based on the cDNA clones (such as EAS or TROP1). Here, we will use the term EpCAM throughout the text as proposed by Baeuerle and Gires (3) for unified nomenclature.

#### Dynamic expression of EpCAM.

Most of the CAMs are present on virtually all normal cells, contrary to EpCAM, which is in mature cells restricted to simple squamous epithelia and some adenomatous epithelia. To maintain the proper organization of multicellular animals, cells need to be able to adhere, to move relative to each other, and to repel and signal to each other. Individual cells fulfill these tasks by communicating across their plasma membranes. During morphogenesis, CAM-mediated interactions provide adhesive forces required for cells to aggregate and form tissues. CAM-mediated adhesions in developing cells are highly dynamic, which provides the fluidity required for cellular movements that drive morphogenesis. Some CAMs simultaneously act as adhesive molecules and sensors, whereas others have evolutionarily co-opted to function predominantly as adhesive factors or signal-transducing sensors. The exact function of EpCAM is currently being elucidated, but EpCAM appears to play many different roles, including cell–cell adhesion and signal transduction, which appear to be influenced by the spatial and temporal expression pattern of EpCAM.

In normal adult tissues, EpCAM is expressed on the basolateral surface of simple, pseudostratified, and transitional epithelial cells in various tissues of the gastrointestinal tract, reproductive system, and respiratory tract (5, 92). The pattern of EpCAM mRNA expression in tissues is similar to human EpCAM protein, with the highest expression in the gut and lower levels in the kidneys, pancreas, mammary glands, lungs, and genitalia, consistent with its epithelial cell distribution (98). Most normal, nonpathological, epithelial tissue is EpCAM positive, with the exception of epidermal keratinocytes, gastric parietal cells, myoepithelial cells, thymic cortical epithelial, and hepatocytes (92, 111). Interestingly, EpCAM expression has been correlated with many populations of progenitors in the development of organs. This has been demonstrated in the assembly of germ-line cells in development, where EpCAM is expressed during formation and early gonad assembly (1). Neonatal male and female germ cells remain EpCAM positive, whereas EpCAM is absent in adult testis, with the exception of spermatogonia. Similarly, research by Cirulli et al. (21) indicated a regulatory role of EpCAM during morphogenesis of pancreatic islets. Indeed, the authors investigated whether the EpCAM expression pattern observed in the fetal pancreas is retained in the adult pancreas. In contrast to fetal pancreas, the highest levels of expression of EpCAM were not identified in endocrine cells but rather, in small, intercalar; interlobular; and main ducts (21). This developmentally regulated expression and function of EpCAM has also been illustrated in other organs, such as the kidney (130, 131), lung (59), skin (66), and thymus (46).

In mammalian systems, embryonic expression of EpCAM is observed in the initial phases of development, from the fertilized oocyte to the morula. In human and mouse, EpCAM is expressed in embryonic epithelia, but the levels usually drop as cells reach terminal differentiation (129). In this early developmental window, EpCAM expression is not restricted to epithelial precursor cells but is also present in undifferentiated stem cells that are not yet assigned to a specific cell fate (1, 128, 129). Interestingly, in embryoid bodies in vitro, three phenotypically distinct populations of cells could be distinguished with regard to expression pattern of EpCAM: intracellularly or homogenously along the cell membrane or exclusively at restrictive contact points with neighboring cells. Such a distribution of EpCAM in these embryoid bodies may be associated with the differentiation potential of these cells. In later stages of mammalian and zebrafish development, EpCAM expression becomes strictly epithelial specific, and terminally differentiated cells stop expressing EpCAM (120).

In summary, EpCAM expression fades, and its regulatory effects become muted as progenitor cells differentiate through their respective maturational lineages to adult, differentiated fates, accompanied with a drop in stem/progenitor activity, which suggests that the level of cellular differentiation and specialization, at least partly, depends on EpCAM expression (41, 53, 82, 99, 129).

#### EpCAM and cancer.

The role of EpCAM in cancer research has been covered in numerous articles (3, 38, 39, 54, 103, 115, 139) and is beyond the scope of this review; therefore, we will only touch on the insights garnered from work of EpCAM in cancer. Generally, EpCAM is not found on tumors of mesodermal and ectodermal origin but on most, if not all, carcinomas. High expression levels of EpCAM in primary tumors are often associated with proliferation and a more aggressive phenotype with respect to overall survival and appearance of metastases. Actually, EpCAM is the prime epithelial antigen in use to isolate CTCs and to characterize DTCs once they have left the primary cancer. CTCs and DTCs are central intermediates during tumor progression and reflect the tumor's ability to remain in the body, circulate, and colonize new tissue to form lethal metastases. In this respect, reduced expression of EpCAM was also associated with epithelial-to-mesenchymal transition, and recently, a lack of EpCAM on CTCs has emerged and might offer a mechanism by which cells can escape the strict architecture of their tumor (40). These opposing expression patterns in primary tumors vs. CTCs/DTCs might reflect a context-dependent adaption of EpCAM expression or show a difference in EpCAM processing during metastatic progression (30). Thus knowledge on the function of EpCAM in these cells is valuable and requires additional work.

#### EpCAM and its plethora of functions.

**Fig. 2.**
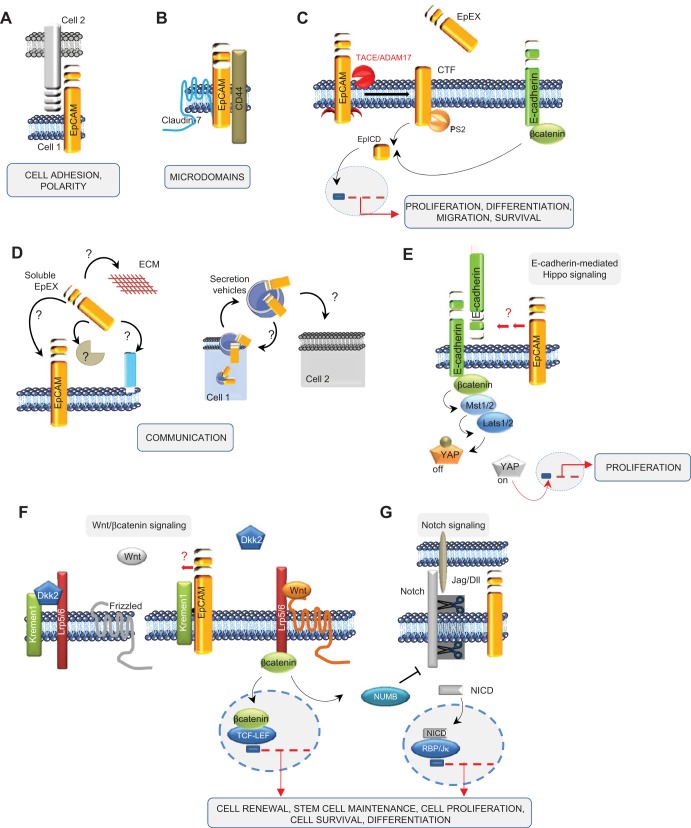
EpCAM and its plethora of functions. EpCAM has been described to play a role in cell adhesion (*A*) and in formation of microdomains (*B*), having its own signaling with the release of its C terminus, EpCAM intracellular domain (EpICD; *C*), and involvement in cell communication (*D*), and interacts with other signaling pathways, such as Hippo (*E*), β-catenin/wingless (Wnt; *F*), and Notch (*G*) signalings. EpEx, extracellular domain of EpCAM; TACE/ADAM17, TNF-α-converting enzyme/a disintegrin and metalloproteinase 17; CTF, COOH-terminal fragment; PS2, presenilin-2; Mst1/2, mammalian sterile 20-like kinase 1/2; Lats1/2, large tumor suppressor kinase 1/2; YAP, Yes-associated protein; Kremen1, Kringle-containing transmembrane protein 1; Dkk2, dickkopf homolog 2; Lrp5/6, lipoprotein receptor-related proteins 5 and 6; TCF-LEF, transcription factor- lymphoid enhancer factor; Jag/DII, Jagged/Delta-like; NICD, Notch intracellular domain; RBP/Jκ, recombination signal-binding protein for Ig κ J region.

The functions of EpCAM are highly diverse and are summarized in Fig. 2, and many of these functions have been discussed in other reviews (115, 129). EpCAM is described as a homophilic cell–CAM, which contributes to tissue integrity (Fig. 2*A*) (45, 72, 77, 93, 98). However, EpCAM is a relatively weak cell–CAM compared with E-cadherin. Whereas able to mediate homophilic adhesion in E-cadherin-negative cells, EpCAM can weaken E-cadherin-mediated intercellular adhesion, suggesting a potential role as modulator of the strength of cell–cell adhesion (76). Furthermore, recent data demonstrated that EpCAM interacts with proteins of the claudin family and fosters tight junction formation and actomyosin-dependent contractility (84, 85). Thus the role of EpCAM in cell adhesion remains somewhat contradictory and would profit from further investigation.

EpCAM has also been found in the copresence of CD44 and claudin-7 in complex molecular networks [called tetraspanin-enriched microdomains (TEMs); Fig. 2*B*] (66, 71, 100). It is believed that claudin-7 pulls EpCAM in TEMs (or differently termed microdomains of the membrane) to foster its activity in metastases and signaling. EpCAM has also been reported to be involved in the regulation of the epithelial integrity by affecting composition, localization, and function of tight junctions via interaction with claudins (147).

Since EpCAM is described on microvesicles, a possible autocrine or paracrine effect is conceivable. Due to its release in its soluble form (soluble EpEx; see below) or presence in microvesicles (e.g., exosomes), potential signals can migrate from cell to cell inside of the same organ or travel around to reach other organs (Fig. 2*C*). Notably, proteomic analysis of exosomes released from colorectal cancer cells has identified the presence of EpCAM on those microvesicles (56, 86), and in bladder cancer, urinary soluble EpCAM levels were detected and increased with stage and grade of the tumor (10). Additional functions have also been assigned to EpCAM, such as regulation of contractility and morphogenic movements and regulation of proliferation (83, 94).

One of the most recent discoveries is the proteolytic cleavage of EpCAM at multiple sites (48, 83, 115). EpCAM undergoes a regulated, intramembranous proteolysis, beginning with cleavage of the EpEx by sheddases of the a disintegrin and metalloproteinase (ADAM) family (14, 83) and by protease β-secretase 1 (BACE-1) (48). Shedding-dependent activation of signal transduction by EpCAM is perpetuated further by cleavage via the γ-secretase complex, allowing the generation and nuclear translocation of the EpICD (Fig. 2*C*). The short-lived EpICD uses components of the wingless (Wnt) signaling pathway (83) and activates promoters of stemness genes (82) and cell-cycle regulators (18) (see Fig. 2*C*). Regulated intramembrane proteolysis (RIP) of EpCAM is a prerequisite for EpCAM-dependent proliferation (24, 83, 94) and regulation of tumorigenic and pluripotency features (74, 82) and additionally, results in degradation of the protein (48). Since Schnell and coworkers (116) have highlighted that EpCAM is proteolytically processed by an additional unknown pathway, the proteolysis seems to be a crucial event in the regulation of EpCAM signaling. Interestingly, a comparison of the use of two antibodies (clones: MOC-31 and 9-2, recognizing the N- and C-terminus domains of EpCAM, respectively) has been carried out recently on human pancreatic cancer (34) and on a comprehensive multitissue microarray (35) to allow discrimination between two variants of the membranous EpCAM form: EpEx^+^/EpICD^+^ (EpCAM^MF^) and EpEx^+^/EpICD^−^ (EpCAM^MT^). The investigators found a high presence of EpCAM^MF^ in noncancerous tissues, whereas in pancreatic cancer, EpCAM^MT^ was highly increased and associated with a more aggressive phenotype (34, 35). These results suggest the presence of two distinct EpCAM variants that may occur during carcinogenesis and that loss of membranous EpICD expression is a frequent event in human cancer.

Besides “physical” contact with other partners at the plasma membrane, EpCAM can interact with different signaling pathways. The Hippo-Yes-associated protein (YAP) pathway mediates the control of cell proliferation by contact inhibition, as well as other attributes of the physical state of cells in tissues (47). One of the upstream-interacting proteins proposed to modulate the core Hippo signaling pathway is E-cadherin (see Fig. 2*E*). Indeed, the group of Gumbiner (64) showed that Hippo signaling pathway components are required for E-cadherin-dependent contact inhibition of proliferation, suggesting that in addition to its role in cell–cell adhesion, E-cadherin-mediated cell–cell contact modulates the Hippo signaling pathway to control cell proliferation. Since EpCAM interacts with E-cadherin, one can imagine that in return, Hippo signaling can be disrupted, leading to activation of YAP and downstream transcriptional activation (Fig. 2*E*).

The Wnt signaling pathway is a crucial mediator of normal organ development during embryogenesis and tissue repair; consequently, many functions are allocated to Wnt, such as axis patterning, cell polarity, cell-fate specification, cell proliferation, and cell migration. Interestingly, regulatory elements of the *EPCAM* gene promoter are responsive to transcription factor 4 (Tcf4), a downstream effector of the Wnt pathway (149). Owing to the frequent deregulation of the Wnt pathway in cancer cells and to its functions in progenitor cells (107), a concomitant upregulation of EpCAM in these cell types may possibly be mediated by the Wnt pathway. Recently, EpCAM has been described to be a de-repressor of Wnt signaling in an indirect manner (81) (see Fig. 2*F*). In this model, EpCAM reduces the membrane turnover of lipoprotein receptor-related protein 6 (Lrp6) by sequestrating Kringle-containing transmembrane protein 1, a gene encoding a high-affinity dickkopf homolog 1 (DKK1) transmembrane receptor that functionally cooperates with DKK1 to block Wnt/β-catenin signaling. Thereby, EpCAM fosters Lrp6 retention at the membrane and its signaling through Wnt2bb, representing a licensing factor for the endodermal differentiation toward hepatocytes in zebrafish. Importantly, this study describes for the first time a de-repressor role of EpCAM (81).

Recently, it has been described that the balance between Notch/Wnt signaling regulates the commitment of HSPC during liver repair (8, 25, 123). During biliary regeneration, expression of Jagged 1 (a ligand of Notch) by myofibroblasts stimulated Notch signaling in HSPCs and thus their biliary specification to cholangiocytes. Alternatively, during hepatocyte regeneration, macrophage engulfment of hepatocyte debris induced Wnt3a expression, sustaining NUMB expression within HSPCs to promote their specification to hepatocytes (8) (Fig. 2*G*). Thus converging this study (8) with the elegant work of Lu and colleagues (81), one could imagine that EpCAM might represent a route to switch this balance between Notch and Wnt toward Wnt signaling.

## EMERGING CONCEPTS IN THE FIELD OF HSPC BIOLOGY

Nonalcoholic fatty liver disease has become one of the most common forms of chronic liver disease, including a wide spectrum of pathological liver conditions, ranging from simple hepatic fat accumulation to nonalcoholic steatohepatitis, with or without fibrosis, eventually progressing to cirrhosis and hepatocarcinoma (50). Liver transplantation has been thought to be the only convincing therapy to deal with end-stage liver disease, although it has certain disadvantages, including a high risk of rejection. This concern has led to identification and isolation of a source of stem-cell subpopulations with a high potential to generate a large amount of hepatocytes for repairing the damaged liver. The quest to identify resident stem cells in the liver and to isolate and expand them with high efficiency remains challenging, and the understanding of their biology remains in its infancy compared with other stem-cell tissues, such as intestine or skin. However, some consensuses have been reached and are discussed below. Further discussion of the data can be found in further reviews (6, 27, 31, 36, 55, 89, 91, 106, 126, 140) and references therein.

**Fig. 3.**
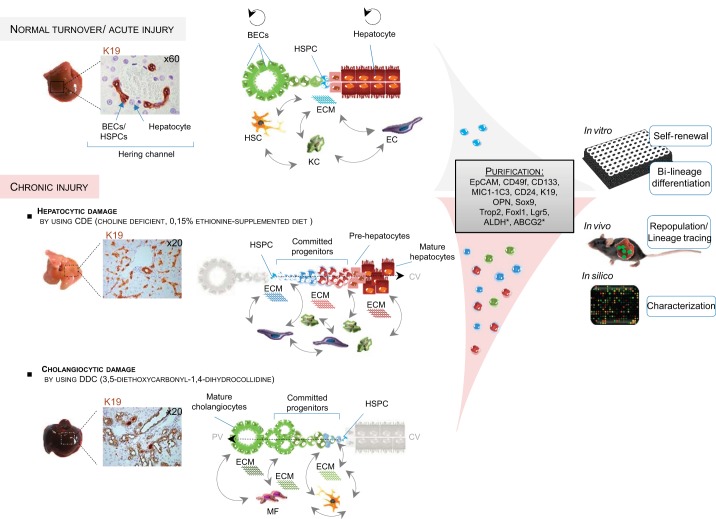
Basics on hepatic stem/progenitor cells (HSPCs) from adult liver under normal and injured conditions. In healthy livers, mature cells are taking care of the normal homeostasis of the liver without intervention from the stem-cell compartment. The latter is located in the channel of Hering, illustrated by K19 positivity (cytokeratin 19). The niche cells [hepatic stellate cell (HSC), Kupffer cell (KC), endothelial cell (EC)] preserve the stemness of this compartment. Upon severe injury, HSPCs emerge and expand (observed by an increased number of K19^+^ cells), contributing to liver repair. Depending on the nature of the insult [hepatocytic vs. cholangiocytic, determined by use of different diets: choline-deficient, ethionine supplemented (CDE) vs. 3,5-diethoxycarbonyl-1,4-dihydrocollidine (DDC), respectively], niche hepatic cells and ECMs are involved differently, supporting the HSPC fate. HSCs/myofibroblasts (MF) and collagen deposition are associated with HSPCs in biliary regeneration [biliary epithelial cells (BECs)] and KCs with hepatocytic regeneration. Isolation/purification by cell sorting (based on differential marker expression or functions) and subsequent cell culturing demonstrate the “plasticity” of those cells, containing a subpopulation with stem-cell features, which are defined in vitro by clonogenicity and bilineage differentiation potential and/or in vivo by transplantation assays. Genetic-lineage tracing is used to identify the fate or the origin of these HSPCs, whereas “omics” analysis is used for their characterization. PV, portal vein; CV, central vein; MIC1-1C3, macrophage inhibitory cytokine-1-1C3; OPN, osteopontin; Sox9, sex-determining region Y-box 9; Trop2, trophoblast cell-surface antigen 2; Foxl1, forkhead box l1; Lgr5, leucine-rich repeat-containing G protein-coupled receptor 5; ALDH, aldehyde dehydrogenase; ABCG2, ATP-binding cassette subfamily G, member 2.

In both normal turnover of the hepatic tissue and acute disease, the liver predominantly activates terminally differentiated epithelia (i.e., biliary epithelial cells and hepatocytes) to proliferate and repair (Fig. 3). In chronic and severe injury, however, this capacity fails, and ductular reactions of activated biliary epithelial cells that contain putative stem/progenitor cells appear in the periportal region of the liver to restore both hepatic architecture and synthetic liver function. Generally, stem-cell niches are dynamic cellular and extracellular microenvironments that balance stem-cell activity to preserve tissue homeostasis and repair throughout the lifespan of an organism (63, 141). In healthy liver, the complete nature of all components of this niche remains unclear; unlike in the intestine, the cellular and acellular components of the microenvironment remain poorly described. Three-dimensional reconstructions in human liver suggest that the progenitor microenvironment arises from the interface between the hepatocyte canalicular system and the biliary tree (positive for keratin 19), known as the canals of Hering (127), which was also identified as the main facultative niche in the mouse (67) (see Fig. 3). The HSPC niche is composed of different cell types (including hepatic stellate cells, Kupffer cells, endothelial cells) (79), ECM components, growth factors, and cytokines released by the niche cells to help and maintain the characteristics of HSPC and the balance among their activation, proliferation, and differentiation (Fig. 3).

Attempts to identify putative HSPCs have assessed the ability of these cells to differentiate toward both hepatocytic and biliary lineages, as well as their clonogenic capacity to look at their stem-cell potential (Fig. 3) (29, 118). Alternatively, the regenerative capacity of HSPCs can be shown in vivo using liver-repopulation assays (124, 142) or direct lineage tracing. A number of HSPC markers have been proposed, but none are completely specific (42). Several proteins or activities that discriminate the HSPCs from their surrounding cells exist; EpCAM (112, 113, 154), Prominin1 (or CD133) (108), and macrophage inhibitory cytokine-1-1C3 (29) antibodies or a combination of these have been used to enrich for these cells, whereas activities that are enhanced in HSPCs, such as efflux transporter activity [e.g., side-population technique (43) or aldehyde dehydrogenase activity (26)], are more intricate but can also be used to isolate HSPCs.

The development of strategies to perturb niche components has provided insight into the responsive nature of the niche and offers a framework to uncover how disruption of a normal stem-cell niche function may contribute to the onset of the ductular reaction and its progression. For such purposes, different liver-injury mouse models have been used to study HSPC activation, and they are reviewed elsewhere (27, 78, 102). Figure 3 illustrates two different treatments: 3,5-diethoxycarbonyl-1,4-dihydrocollidine and choline-deficient ethionine supplemented, which are often used to activate HSPC.

In fetal and neonatal livers, the HSPCs are concentrated in ductal plates, whereas in pediatric and adults livers, the canals of Hering are their principal niches (15, 67, 127, 155). It should be noted that the canals of Hering are the adult remnant of the ductal plates containing the bipotent HSPCs. Whereas the HSPC population is high in number in fetal livers, its number drops drastically in adult livers. Furthermore, it has been shown that the liver is similar to other tissues, in that regenerative responses in postnatal tissues parallel those occurring in development (27, 28, 42, 155).

Furthermore, the liver is comprised of a maturational lineage of cells that represents all maturational stages, from naïve cells in the niche of the canal of Hering, located periportally, progressing through the midacinar region, and ending with the most mature cells found pericentrally (134). The best-described maturational lineage is the hepatocytic; the second lineage (cholangiocytic) is still in the process of being defined. Within the hepatocytic maturational lineage, the parenchymal cells interact continuously with their mesenchymal cell partners (134). The understanding of the intermediate stages and cues that regulate maturational lineage in HSPC biology, as well as the symmetry between fetal development and adult regeneration, represents a major breakthrough in the field.

Localization of the liver epithelial compartment and those cells with which it interacts in its microenvironment determine HSPC specification, and the resulting changes within the canals of Hering and adjacent cells are a consequence of local signaling cascades (8, 9) (see Fig. 3). Indeed, cell–cell signaling from both hepatic stellate cells and macrophages can influence HSPC fate via the Wnt and Notch pathways following liver injury (77). In hepatocellular regeneration, the progenies of HSPCs irradiate from the portal tracts in which they are enclosed in laminin, which facilitates their expansion. Upon exit from the laminin niche, these cells are subject to differentiation cues, such as Wnt and hepatocyte growth factor, which activate the prohepatocyte transcriptional cascade in hepatic progenitor cells. In biliary regeneration, HSPCs emerge in a similar manner, but they remain in the laminin ECM, in which fibroblasts are able to influence their maturation through activation of the Notch signaling pathway. This pathway influences the activation of the hepatocyte nuclear factor (HNF)6/HNF1β transcriptional network to specify cholangiocytes correctly (8, 9).

The regenerative response in adult liver involves a heterogeneous population of cells containing a range of cell types from primitive progenitors to more committed hepatocyte-like cells, illustrating different cell-fate transitions and lineage reprogramming. Interestingly, during this reprogramming, the ductular cells express particular markers that are useful for progenitor cell isolation [such as forkhead box l1 (Foxl1), TROP2, neural CAM (N-CAM), Delta-like 1 homolog, neighbor of Punc E11, and leucine-rich repeat-containing G protein-coupled receptor 5]. Nevertheless, the function of these molecular markers remains obscure. The trigger for the formation of the ductular reaction and the mechanisms controlling migration of HSPCs or the detachment of cells from the cellular or acellular components of the niche upon liver repair are not well understood. One could imagine that the molecular switches may play a significant role during liver development and regeneration. Likewise, additional molecular switches have been observed already and might regulate redistribution of some metabolic functions throughout the hepatic lobule (e.g., gluconeogenesis, fatty acid oxidation, glutamine synthetase) or deal with the protein responsible for the maintenance of the hepatic zonation (e.g., β-catenin, E-/N-cadherins) upon liver injury and repair.

Recently, two elegant reports have investigated the potential importance of this cadherin-based molecular switch with respect to the modulation of the formation of ductular reaction in liver injury (117, 132). In normal liver, N-CAM marks biliary cells and HSPCs but not mature hepatocytes. N-CAM mediates cell–cell adhesion by multiple modes, including homo- and heterophilic interactions and cell–matrix contacts. These interactions are modified by the post-translational modification of N-CAM with polysialic acid (or polySial-NCAM). Due to the size of the polysialic acid chains on N-CAM, N-CAM functions change from adhesive to antiadhesive (57). Whereas in normal liver, N-CAM contributes to the stable settlement of HSPCs, subsequent to injury, polysialic acid is produced and changes N-CAM function by weakening cell–cell and cell–matrix interactions, facilitating the migration (132). During differentiation into hepatocytes, polySial-NCAM and N-CAM are cleaved from the cell surface of the HSPC descendants, reducing migration of ductular reaction into parenchyma but allowing their hepatocyte differentiation (132). Previously, it has been demonstrated that Foxl1 is a marker of HSPCs found in the injured liver and that these Foxl1^+^-tagged cells could be isolated, expanded, and differentiated toward the cholangiocyte and hepatocyte lineages in vitro (109, 118) and in vivo (117). Recently, the authors demonstrated that specific ablation of Foxl1-positive HSPCs and their descendants impair recovery of the liver from toxic injury, illustrating that the cells marked by Foxl1 are required for development of both lineages (117).

Obviously, HSPCs have the potential to provide patients with a comprehensive and most biological robust regeneration and might also be able to repopulate exhausted niches in the regenerating liver. To this end, detailed knowledge of the biology of HSPCs, particularly the understanding of molecular switches involved in processes of HSPC maintenance and differentiation into mature liver cells, is necessary. Only with the identification of these molecular switches can we hope to design rationally compounds against these signals to enhance the regenerative process in the adult liver.

## EpCAM AND ITS MULTIPLE FACETS DURING LIVER DEVELOPMENT, HOMEOSTASIS, AND REGENERATIVE RESPONSES

Normal epithelia express EpCAM at a variable but generally lower level than carcinoma cells (5, 128, 129). It is only recently that experimental approaches on EpCAM function shifted to nonmalignant cells and to the potential role(s) of EpCAM in normal morphogenesis and organogenesis (128, 129). With respect to liver, studies from Reid and collaborators (112, 113, 134, 155) have investigated the histological location of EpCAM in human livers from fetal to adult donors. In fetal livers, EpCAM expression was found in the ductal plate cells as well as in parenchymal cells, throughout the developing hepatic lobule (155). EpCAM was expressed most abundantly in ductal plate cells, where membranous and cytoplasmic staining was evident. By contrast, expression of EpCAM was restricted to a membranous pattern in human hepatoblasts. In neonatal livers, EpCAM expression is similar to that observed in fetal livers, in that there is a recognizable expression in ductal plates around portal tracts and a lack of expression of EpCAM in mature hepatocytes (112, 113, 155). In pediatric livers, the ductal plate is no longer apparent. Rather, one observes canals of Hering near the portal triads, which are surrounded by mature hepatocytes. Interestingly, human hepatoblasts are tethered to the ends of the canals of Hering, with EpCAM expression exclusively membranous, whereas the remnant ductal plate cells (forming the actual Hering canals at that stage) are expressing EpCAM in the cytoplasm and the plasma membrane. In adult liver, biliary epithelial cells/HSPCs (either forming the bile ducts or the canals of Hering), but not hepatocytes, are found to express EpCAM (112, 113, 155). EpCAM expression was cytoplasmic and membranous in the interlobular bile duct, ductules, and canals of Hering. The canals of Hering are strongly and intensively positive for EpCAM (and cytokeratin 19). Human hepatoblasts with membranous staining for EpCAM localize near the canals of Hering and are sometimes found tethered to the ends of the canals of Hering. The percentage of human EpCAM-positive hepatoblasts declines rapidly after birth, such that the hepatoblasts constitute <0.01% of the parenchyma in pediatric and adult livers. These findings, elegantly described in human liver sections (112, 113, 155), match with those established previously, in which the percentages of human hepatoblasts and HSPCs were defined by flow cytometric analyses (113).

Complementary studies using murine hepatic tissues showed that hepatoblasts exhibit an analogous, dynamic expression of EpCAM during liver development. Early studies conducted by de Boer et al. (23) in healthy adult livers illustrated that EpCAM was found exclusively on biliary epithelial cells and HSPCs, although it was present in the great majority of fetal liver hepatocytes of 8-wk-old embryos. Later, the group of Miyajima (125) investigated the expression of EpCAM at different stages of mouse liver development. They found that EpCAM was highly expressed at the onset of fetal liver organogenesis and decreased gradually along with hepatic differentiation to the point where it was restricted to biliary epithelial cells/HSPCs but entirely lacking in hepatocytes at later stages (125). Tanaka and colleagues (101, 125) were then the first group to show that murine EpCAM is the earliest and a transient marker for hepatoblasts during hepatogenesis. Furthermore, in in vitro studies, different groups reported that EpCAM is expressed on HSPCs isolated from mouse and rat (26, 112, 153, 154), and upon in vitro differentiation conditions, those cells give rise to hepatocytes that are devoid of EpCAM expression (61, 63, 66, 81–83). In addition, when freshly isolated human EpCAM-positive cells (from either fetal or postnatal livers) were transplanted to nonobese diabetic/severe combined immunodeficiency (NOD/SCID) recipient mice, they engrafted and repopulated the liver tissue and lost EpCAM expression (113).

Pathological changes of the liver are accompanied by a strong re-expression of EpCAM, for example in hepatocellular carcinomas, where EpCAM serves as a marker for cancer stem cells (151). Indeed, Yamashita and colleagues (151) have shown that EpCAM^+^/α-fetoprotein tumor cells display hepatic cancer stem-cell traits, including the abilities to self-renew and differentiate and to be able to initiate highly invasive hepatocellular carcinoma in NOD/SCID mice. Upon massive liver necrosis, regenerative responses involve the expansion of human EpCAM-positive HSPCs, whereas no additional EpCAM positivity in any other cell types is observed (155). In contrast, regeneration in biliary cirrhosis involves human hepatoblasts with a membranous expression of EpCAM, concomitant to the expansion of human HSPCs (155). During liver injury (chronic hepatitis C/B), EpCAM marks those hepatocytes, freshly derived from stem cells, but is lacking on those derived from pre-existing hepatocytes, thus suggesting an association of EpCAM with multipotent progenitors (152). Indeed, hepatocytes that are positive for EpCAM are located close to ductular reactions and have a longer telomere length than EpCAM-negative hepatocytes, suggesting their origin from a slow-cycling HSPC.

Taken together, data obtained from both mouse and human tissues suggest that hepatocytic differentiation is accompanied by a transition from an EpCAM-positive to an EpCAM-negative state of cells, which mostly recapitulates essential stages of embryonic hepatogenesis. Consequently, the deciphering of the association between EpCAM expression and its functions by focusing on how it is regulated with regard to HSPC activation may represent a new axis for understanding HSPC biology.

## A COMPLEX MECHANISM TO SWITCH EpCAM EXPRESSION

### 

#### Membranous or RIPed EpCAM.

**Fig. 4.**
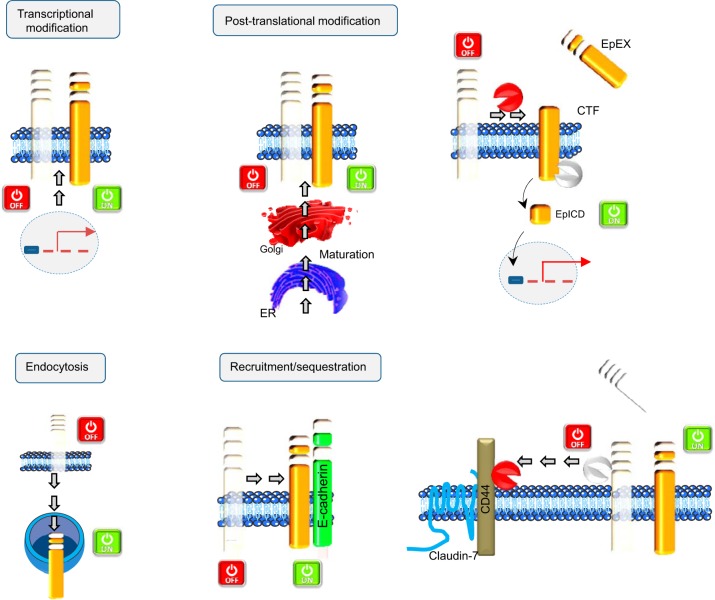
Mechanisms regulating EpCAM. Four different categories of mechanisms have been illustrated for explaining the regulation of EpCAM: transcriptional modification; post-translational modification, which implies its glycosylation status or the regulated intramembrane proteolysis (RIP); endocytosis; and recruitment/sequestration. ER, endoplasmic reticulum.

**Fig. 5.**
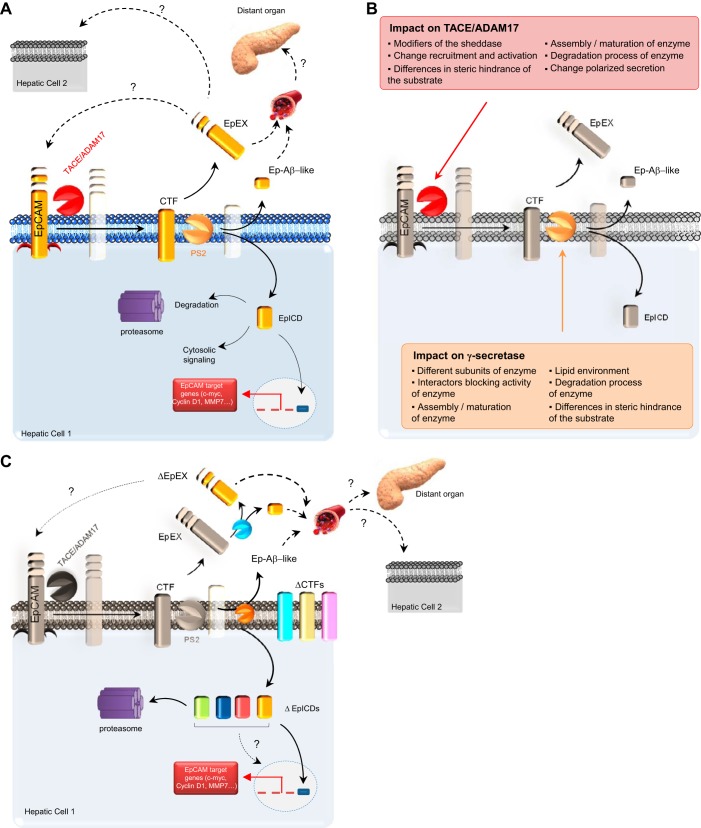
Ectodomain shedding and RIP: important post-translational modifications bringing diversity to the EpCAM function. *A*: cleavage within the transmembrane domain of EpCAM results in shedding of the EpEx and accumulation of the EpICD to the nucleus. Since EpCAM is also described on microvesicles, a possible autocrine or paracrine effect is likely. Ep-Aβ-like, small EpEx fragment; MMP7, matrix metalloproteinase-7. *B*: enzymes responsible for these 2 events are TACE/ADAM17 (red “Pac-Man”) and γ-secretase (tan Pac-Man), indicating that playing with the function/expression of these proteases adds diversity to the function of EpCAM. Some possibilities are listed in the text boxes, respectively, for TACE/ADAM17 and PS2. *C*: EpCAM can be cleaved at multiple positions within its ectodomain, resulting in various N-terminal proteolytic fragments given birth to variant (Δ) CTFs. Moreover, variants of EpICD have recently extended our restrictive view of EpCAM, as to its potential impact in cell signaling. Soluble EpEX can also be cleaved by ADAM proteases and BACE1 (blue Pac-Man) to release DEpEX and small fragments. For supplemental information, see reviews (49, 51, 69, 73, 88).

The accumulation of data on the biology of EpCAM has offered explanations on how to switch EpCAM on and off at the functional or expression level. Furthermore, increasing evidence indicates that subcellular localization of EpCAM is critical. These fine-tuned regulatory mechanisms may control the distribution of EpCAM and thereby its functionality as a de-repressor of hepatocytic differentiation and a pluripotency-associated factor. A general overview of these potential mechanisms is summarized in Figs. 4 and 5.

Transcriptional modifications are main mechanisms used to switch on/off any gene; hypothetically, one could imagine that similar scenarios are happening as well for EpCAM. Both methylation of DNA at cytosine residues within CpG islands as well as trimethylation of lysine residues of histone 3 have been reported to impact the transcription of the *EPCAM* gene in cancer and human embryonic stem cells, respectively (82, 121, 138). Besides these epigenetic chromatin modifications, post-translational changes are also reported to influence the expression of EpCAM. For instance, glycosylation of EpCAM is required for prolonged plasma membrane retention (Fig. 4). Indeed, three independent N-glycosylation sites at asparagine residues N^74^, N^111^, and N^198^ in the EpEx part dictate the half-life of EpCAM at the cell surface. Especially, mutation of asparagine in position 198 resulted in a severely reduced retention of EpCAM at the plasma membrane from ∼21 to 7 h (95). The regulation of levels and composition of glycosylation might impact the subcellular location and stability of EpCAM. Although it has never been demonstrated experimentally, results from therapeutic antibodies and cleavage studies suggest that endocytosis is an additional means by which the EpCAM expression can be regulated (Fig. 4); e.g., the killing of cells using toxin-conjugated EpCAM-specific antibodies is a long-accepted therapeutic option (119) despite a formal lack of proof of endocytosis of EpCAM. Along the same line, cleavage of murine EpCAM was reported to be fulfilled by ADAM proteases at the plasma membrane but additionally, by the β-secretase BACE-1 (48). However, BACE-1 is active at a pH optimum of 4.5 and therefore, requires the acidic environment of endo- and lysosomes, hence suggesting the endocytosis of murine EpCAM.

The pleiotropic functions of EpCAM can be allocated to the full-length protein, as well as to EpCAM-derived fragments, which are generated upon RIP. Dynamic signaling through EpCAM not only requires the presence or absence of full-length EpCAM at the cellular membranes but also is contingent on the varying rates of the formation of EpCAM-derived fragments that have their own regulatory properties and in changes in the association of EpCAM with interaction partners (Fig. 4). Generation of biologically active proteins by RIP represents a fascinating strategy for cellular signaling, which is highly conserved from bacteria to humans. This mechanism is involved, not only in degrading membrane-spanning segments (also termed the membrane proteasome) but also, in generating messengers that elicit biological responses (73). The first cleavage of EpCAM results in shedding of its ectodomain (EpEx; Fig. 5) and can be conducted by at least two types of secretases: i.e., λ- and β-secretase. The second cleavage, which is strictly dependent on the first, occurs within the transmembrane domain, resulting in secretion of a small peptide (Ep-Aβ-like) and the release of the EpICD into the cytosol. In human carcinoma cells, EpICD can translocate further into the nucleus and act as a signaling molecule to regulate the transcription of target genes. The RIP itself is tightly regulated (51, 73), and indeed, cellular processes affecting the recruitment, activation, or polarized secretion of sheddases can influence shedding (Fig. 5).

Altogether, proteins involved in the retention of EpCAM in intracellular or membranous compartments, as well as mechanisms orchestrating RIP of EpCAM, need further investigation.

#### EpCAM expression upon liver repair.

**Fig. 6.**
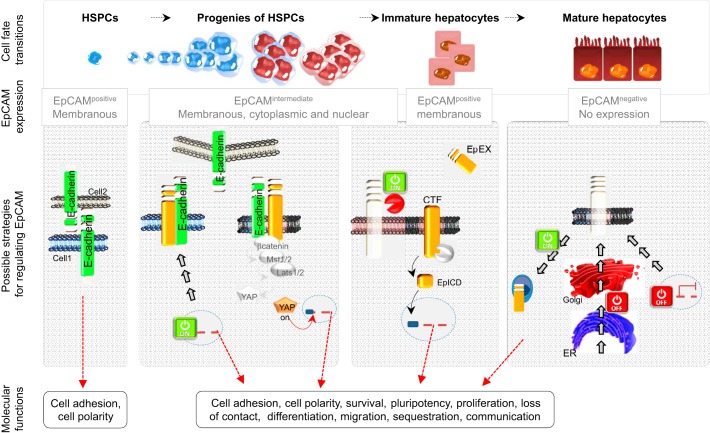
Illustrations of the hypothetical EpCAM-related molecular events occurring in liver regeneration and their relationship to hepatobiliary differentiation. *Top*: EpCAM displays a dynamic expression upon liver repair and is associated with EpCAM-positive cells within so-called ductular reactions (HSPCs and their progenies), which gradually lose the expression of EpCAM along with maturation into hepatocytes. *Bottom*: possible scenarios illustrating the mechanisms regulating EpCAM, followed by their corresponding molecular functions.

With the consideration of the pleiotropic functions of EpCAM (Fig. 2), it is not surprising to find that the promotion of its sustained localization at the plasma membrane or favoring of its retention in the cytoplasm by endocytosis—accelerating the degradation of its EpICD variants to prevent any signaling or change its membranous partnership—may quickly change its function and might occur to permit the HSPCs to be released from naivety and to differentiate (Fig. 6). Alternatively, with the weakening of E-cadherin cell–cell adhesion, EpCAM might foster higher cell plasticity within epithelial tissues, which in turn, may help promote motility during the HSPC activation.

It is tempting to hypothesize that proteolytic cleavage of EpCAM may appear as an alternate mechanism to fine-tune the plasticity of EpCAM-expressing cells and that it might influence the fate or the biological activity (proliferation vs. differentiation) of stem/progenitor cells during differentiation and regeneration (Fig. 6). The use of a γ-secretase inhibitor {i.e., N-[N-(3,5-difluorophenacetyl)-l-alanyl]-S-phenylglycine t-butyl ester} by Boulter et al. (8) in their in vitro and in vivo experiments decreased Notch activity in the HSPCs (this can also be achieved through repression of NUMB) and inhibited accumulation of hepatocytic progenitors, whereas it enhanced outgrowth of progenitors with a biliary phenotype. Whereas Notch signaling inhibition was the target, EpCAM signaling could be influenced as well in this experimental setting, since EpCAM also belongs to the same RIP substrates as Notch (69). The observed differentiation events, induced by γ-secretase inhibition, might therefore reflect cleavage inhibition of Notch but also EpCAM and even other RIP family substrates.

On the other hand, alternative considerations on this transient change of EpCAM expression might be due to additional scenarios that would need further investigation and are briefly described as follows.

Although the liver is a central metabolic organ in adults, it mainly functions as a transient site for definitive hematopoiesis in fetuses. Although the majority of liver metabolic functions appears peri- or postnatally, hematopoiesis-supporting activity of the liver is lost during late-fetal development. Finally, hematopoietic cells move into the bone marrow around the perinatal stage to constitute the adult-type hematopoietic system. With the consideration of the clear change of liver function from these two developmental stages, in which the radical change of hepatic EpCAM expression occurs, it would be rational to observe that the presence of EpCAM on the precursors of hematopoietic cells may play an important role during liver development and may be an important clue in fetal hematopoiesis. One could imagine that EpCAM might be used as a single anchoring protein for the newly synthetized, immature hematopoietic cells, allowing them to reside within a niche in this transient organ before migrating into the bone marrow at the adult age, when hepatocytes lose EpCAM expression. It has been demonstrated that bone marrow erythroid progenitor cells express EpCAM (32), and interestingly, hematopoietic cells synthetize high levels of oncostatin M, which is a strong inducer of the maturation of the hepatocyte lineage (90) and in turn, terminates embryonic hematopoiesis, promoting relocation of hematopoietic cells (65). Since the symmetry between fetal development and adult regeneration has been described and with the knowledge that the ductular reaction is invariably accompanied by inflammatory cells recruited from the bone marrow, the re-expression of EpCAM on HSPC descendants in adult liver might also reflect a particular relationship between the two cellular compartments. In fact, recent work by Bird et al. (7) has demonstrated that the exogenous application of mature bone marrow-derived macrophages is sufficient to induce HSPC proliferation in the absence of any underlying disease pathology, once again, reiterating that inflammation and epithelial repair are intrinsically linked.

Another striking comparison relates to the expression pattern of EpCAM in the colon, where EpCAM is expressed in stem cells at the base of crypts but in contrast to the liver, remains expressed in fully differentiated colon mucosa (148). In a small cohort, cleavage of EpCAM and nuclear translocation of EpICD, which was associated with the induction of proliferation, were seen primarily in colon carcinomas and not in normal mucosa (83). Contrary to what is seen in the liver, colon epithelium displays a constant turnover, which might be, in parts, driven by the option to activate EpCAM signaling on the plasma membrane. Retention of EpCAM on the cell surface of differentiated cells might thus allow for a rapid, EpCAM-dependent response when necessary.

Another conceivable scenario for the function of EpCAM in liver repair is that HSPCs may require the (re-)expression of EpCAM at their membrane to mature successfully and restrictively toward the hepatocytic lineage in response to the niche cell signals (8, 81). Although mesenchymal stromal and cellular partners are the chief orchestrators for these cell-fate decisions (8), EpCAM might confer the ability to translate the signal that progenitors sense from their neighborhood by regulating the bioavailability of receptors (i.e., sequestration of corepressor), as was shown for Lrp6 by Lu et al. (81). In such a scenario, the presence of EpCAM on the plasma membrane in developing hepatocytes would foster Wnt signaling to differentiate further toward hepatocytes instead of biliary tract cells (Fig. 6). The unraveling of EpCAM interactors that allow higher-order ligand-receptor complexes at the plasma membrane to fine-tune their functions, is therefore of great importance.

CAM-mediated adhesion may indirectly promote receptor/ligand signaling by bringing the plasma membranes of opposing cells together and forcing interaction between a ligand on one cell and its receptor on the other cell. In particular, EpCAM has been found to co-purify with glycolipid-enriched lipid microdomains (22, 68, 114) that have the potential to organize signaling complexes at the cell surface. In that context, one can imagine that once located (or redirected by some mechanisms) in those microdomains, EpCAM might easily interfere with important signaling pathways, such as those for Wnt, Notch, and Hippo.

## EpCAM ON PROGENITORS OF THE PERIBILIARY GLANDS

Recently, peribiliary glands have been described to hold immature HSPCs in the intra- and extrahepatic biliary trees. The description of the latter compartment is beyond the scope of the review but comprehensively reviewed elsewhere (11–13, 17, 144). The notion of EpCAM driving liver differentiation is in line with recent data provided by Reid and collaborators (12, 13) concerning biliary tree stem cells. Multiple populations of very primitive stem cells have been identified in the peribiliary glands of the biliary tree, located near the fibromuscular layer and in especially high numbers in the hepatopancreatic common duct. These primitive stem-cell populations have all of the transcription factors required for both liver and pancreas development and high-level expression of pluripotency genes. Surprisingly, they do not express EpCAM. Expression of EpCAM is found only in cells in the peribiliary glands that are intermediate in position between those near the fibromuscular layers and those at the lumen of the bile ducts, a site where markers for mature cells are expressed. Moreover, EpCAM is expressed on cells progressing toward liver and pancreas (144), indicating that its expression does not discriminate hepatic from pancreatic fate but rather indicates that cells are at an intermediate stage of differentiation from HSPCs to mature cells. One could speculate that the absence of EpCAM in multipotent stem cells from peribiliary glands may prevent the occurrence of maturation or commitment to hepatic (or pancreatic) lineages in the peribiliary gland niches, where they are distant from sites of actual hepatic or pancreatic injury, unlike the intraorgan stem-cell niches in close proximity to those sites.

## PERSPECTIVES OF EpCAM IN CLINICAL USE AND REGENERATIVE MEDICINE

Due to a shortage in the supply of human donor organs, adult (and fetal) HSPCs are at the forefront of cell-therapy applications, and more particularly, the use of EpCAM positivity on those particular cells offers an enormous impulse in the regenerative medicine field. Briefly, these include the following details.

*1*) The yield of harvesting the EpCAM^+^ cells is highly efficient from adult livers (∼1,000,000 cells/g tissue) (2, 113), particularly if we consider these cells as potential candidates for liver cell therapies in patients with diverse liver conditions. EpCAM^+^ cells have been found in human livers of all donor ages and pathologies, indicating that EpCAM is likely to be an appropriate and efficient marker to obtain cell suspensions enriched in HSPCs.

*2*) EpCAM^+^ cells successfully repopulate the liver, as Reid and associates (113, 143) have demonstrated. Purified human EpCAM^+^ HSPCs from fetal or postnatal livers are able to engraft the livers of immunocompromised hosts (with or without prior injury) and to give rise to mature human liver parenchymal cells.

*3*) Resistance to hypoxia and ischemia. Adult EpCAM^+^ HSPCs, isolated from human livers when exposed to ischemia, were viable and when cultured on plastic dishes, were able to form colonies of rapidly expanding cells, confirming their extraordinary tolerance to ischemia and illustrating the possibility of using liver samples from a non-heart-beating donor (2, 58, 122, 145).

*4*) Improvement of cryopreservation strategies. In cell therapy, the biggest challenge is to have cells readily available for clinical applications, which relies on unpredictable parameters: acquiring fresh donor organs at the right time. Accordingly, procedures have been established to develop effective cryopreservation protocols before transplantation, allowing cells to be stored for use as an off-the-shelf product whenever the demand is required. A study has provided a successful method for cryopreservation of EpCAM^+^–HSPCs based on the use of hyaluronan-supplemented buffers (135), where the retention of their stem-cell phenotypic traits (i.e., expansion ability, adhesion, and colony formation) has been controlled.

*5*) Improvement of delivery and retention of transplanted cells into the liver. In cell therapy, another major issue is the incapacity of controlling the transplanted cells to home into the organ of interest (105, 133, 135, 137). A way of achieving cell retention involves “grafting strategies,” by embedding cells in biomaterials that concentrate cells in the target tissue, providing a beneficial microenvironment (80). Hyaluronan-based grafts containing EpCAM^+^ cells radically improved the engraftment efficiency over current cell-transplantation approaches (136). Of importance is that grafting significantly reduced the extent of ectopic cell distribution.

*6*) Improvement in analysis of hepatic targeting and biodistribution of HSPCs by using EpCAM^+^ cells. Cell-targeting and engraftment of transplanted cells in desired organ compartments are critical. Therefore, appropriate and noninvasive means of determining the distribution of HSPCs will benefit cell-therapy applications. Lately, indium-111-oxine labeling of human fetal EpCAM^+^ progenitors has been described to be a successful method to assess the targeting of transplanted cells into the liver (19), preferentially when combined with intraportal transplantation. Complementary data, elegantly illustrated by McClelland et al. (87), present a promising method for in vivo cell MRI tracking, enabling noninvasive monitoring of the HSPCs after transplantation.

*7*) Use of anti-EpCAM antibodies in cancer research. Since EpCAM is a pan-epithelial differentiation antigen that is expressed on almost all carcinomas and most importantly, localizes at the cell surface, it has been an attractive target for therapeutic applications in cancer treatment (54, 103). So far, several strategies have been deployed to treat cancer using EpCAM targeting, including diabodies, MAb, and trifunctional antibodies, with the most well-known being Catumaxomab, Edrecolomab, and Adecatumumab, but so far, the results are disappointing (33, 97).

*8*) Use of other tissues for acquiring EpCAM^+^ stem/progenitor cells. With their discovery in the extrabiliary tree tissues (11–13, 16, 17), additional resources of EpCAM^+^ cells are now available. Importantly, in in vitro-culturing conditions, these cells demonstrate clonogenic expansion with maintenance of stemness and also generate mature cells of hepatobiliary and pancreatic endocrine lineages.

*9*) Use of EpCAM^+^ stem/progenitor cells for other purposes. With their potential to generate pancreatic endocrine cells (12, 13, 16, 144), the EpCAM^+^ cells offer hope for saving diabetic patients (61).

## CONCLUSION

Although the strict molecular footprint of hepatic stem cells still remains vague (27), the newly described roles of EpCAM seem to indicate further, broader functions of that protein in endodermal stem cell biology rather than merely being a simple tool for their identification and isolation. Critical appraisal on its biology has illustrated different aspects of EpCAM, and compelling data indicate that different mechanisms exist to switch on/off EpCAM expression or function. Interestingly, an involvement of the EpCAM cleavage products, such as EpEx, the Ep-Aβ-like fragment, and EpICD, in the transcriptional and post-translational regulation of molecules is essential in liver homeostasis or is conceivable in response to injury and merits further attention. The understanding of the functions of EpCAM in the activation of stem cells within the biliary tree and their progression toward a liver or pancreatic fate are parts of a journey that has just begun, but already, additional research in this area is justified, given the growing experimental and clinical data supporting a role for the pathway in regulating outcomes of liver injury (26, 44, 60, 62, 70, 113, 152).

## GRANTS

Support for the work was provided by Interuniversity Attraction Poles (IAP), Phase VII, Contract P7/47 (Federal Science Policy, BELSPO; 10/2012-09/2017); Hepstem: IWT-SBO Contract 090066 (Flemish Government; 11/2009-10/2013); and Brustem: Impulse Program 2011-IP-LS-104, Brustem (Brussels government; 06/2011-05/2014; to L. Dollé and L. A. van Grunsven). In addition, support was provided by Deutsche Forschungsgemeinschaft (GI 540-3/1), Förderung von Forschung und Lehre (Ludwig-Maximilians-University Munich), and Wilhelm-Sander-Stiftung (2009.083.1; 2012.051.1; to O. Gires); National Heart, Lung, and Blood Institute (R01HL108631) and University of Pittsburgh Medical Center (to E. Schmelzer); and Leverhulme Trust and the Medical Research Council (to L. Boulter).

## DISCLOSURES

The authors who have taken part in this study declared that they do not have anything to disclose regarding funding or conflict of interest with respect to this manuscript.

## AUTHOR CONTRIBUTIONS

Author contributions: L.D. and N.D.T. conception and design of research; L.D. prepared figures; L.D. and O.G. drafted manuscript; L.D., N.D.T., E.S., L.B., O.G., and L.A.v.G. edited and revised manuscript; L.D., N.D.T., E.S., L.B., O.G., and L.A.v.G. approved final version of manuscript.
